# The Contrast

**Published:** 1859-12

**Authors:** 


### THE CONTRAST.

A good man says he knew two families who lived within a few minutes walk of each other, both were respectable, and rich; each had seven children, five sons, and two daughters; each lost a son early. The parents of one were eminently pious, and gave to their children a strictly religious education, requiring them to work hard while pursuing their studies; the parents of the other were fashionable people, the "elite" of the village, they were generous, honorable, and high minded, their children were petted, and indulged, and knew no family altar.

A third of a century has passed away, and all the parents have gone to their long home. Those of one family living to fatuity and garrulous old age, their wealth all gone; the others died with peace and plenty all around them, all their surviving children living in abundance, and usefulness, and honor; three of the four sons being ministers of the gospel, working, faithful men; two others are officers in the church, to which they are morally and pecuniarily pillars. Of the other family, only one daughter survives; two sons blew their brains out, another cut his throat, after having killed several men at intervals of several years, and having been a long time the terror of the town.

As a means then of physical, as well as moral health, a consistent religious training is of the first importance, literally giving "promise of the life that now is, and of that which is to come." To those, therefore, of our readers to whom we may speak for the last time, this being the closing number of the present volume, and they not choosing to renew, we give this parting advice;—if you wish your children to grow up to be healthy and useful, a comfort and an honor to yourselves, and the support of society, teach them in their youth, how to pray, and how to work, by praying and working with, and for them; because, while the physical industries impart health, the moral teachings restrain, as well as indispose them, to those intemperances and indulgences which destroy the body and debase the soul. Be assured that it is from praying parents that children come who are to be the lights of their age, and the men of the times. As proof, note that of one hundred students at the Theological Seminary at Columbia, South Carolina, ninety-nine received their first religious impressions from praying mothers.

